# Plasma Metabolome Signatures to Predict Responsiveness to Neoadjuvant Chemotherapy in Breast Cancer

**DOI:** 10.3390/cancers16132473

**Published:** 2024-07-06

**Authors:** Alex Ap. Rosini Silva, Marcella R. Cardoso, Danilo Cardoso de Oliveira, Pedro Godoy, Maria Cecília R. Talarico, Junier Marrero Gutiérrez, Raquel M. Rodrigues Peres, Lucas M. de Carvalho, Natália Angelo da Silva Miyaguti, Luis O. Sarian, Alessandra Tata, Sophie F. M. Derchain, Andreia M. Porcari

**Affiliations:** 1 MS^4^Life Laboratory of Mass Spectrometry, Health Sciences Postgraduate Program, São Francisco University, Av. São Francisco de Assis, 218, Sala 211, Prédio 5, Bragança Paulista 12916900, São Paulo, Brazil; alexrosinisilva@hotmail.com (A.A.R.S.); danilo.colivera@gmail.com (D.C.d.O.);; 2 Department of Obstetrics and Gynecology, Division of Gynecologic and Breast Oncology, Faculty of Medical Sciences, University of Campinas (UNICAMP—Universidade Estadual de Campinas), Campinas 13083881, São Paulo, Brazil; 3 Department of Pathology, Massachusetts General Hospital, Harvard Medical School, Boston, MA 02129, USA; 4 Post Graduate Program in Health Sciences, São Francisco University, Bragança Paulista 12916900, São Paulo, Brazil; 5 Laboratory of Experimental Chemistry, Istituto Zooprofilattico Sperimentale delle Venezie (IZSVe), Viale Fiume 78, 36100 Vicenza, Italy; atata@izsvenezie.it

**Keywords:** breast cancer, cancer biology, drug resistance, metabolomics, neoadjuvant chemotherapy response

## Abstract

**Simple Summary:**

Neoadjuvant chemotherapy (NACT) is a pivotal treatment for breast cancer (BC). However, the success of NACT is uncertain and dependent on BC subtype. A valuable tool to identify biomarkers of the response to chemotherapy is metabolomics. We used plasma from BC patients together with clinical data to verify whether certain metabolites present before NACT can predict the responsiveness of the patient. Liquid chromatography–mass spectrometry and untargeted metabolomic analysis were performed. A statistical model was used to predict the response to NACT for samples separated into two sets: training and validation. The results showed 95.4%/93.3% sensitivity, 94.6%/94.7% accuracy, and 91.6%/100.0% specificity for each set, respectively. The compounds correctly classified 94.9% of resistant and 93.7% of sensitive females. The identified metabolites can be considered together with clinical data, allowing for the development of precision medicine strategies that lead to better treatment choices.

**Abstract:**

Background: Neoadjuvant chemotherapy (NACT) has arisen as a treatment option for breast cancer (BC). However, the response to NACT is still unpredictable and dependent on cancer subtype. Metabolomics is a tool for predicting biomarkers and chemotherapy response. We used plasma to verify metabolomic alterations in BC before NACT, relating to clinical data. Methods: Liquid chromatography coupled to mass spectrometry (LC-MS) was performed on pre-NACT plasma from patients with BC (*n* = 75). After data filtering, an SVM model for classification was built and validated with 75%/25% of the data, respectively. Results: The model composed of 19 identified metabolites effectively predicted NACT response for training/validation sets with high sensitivity (95.4%/93.3%), specificity (91.6%/100.0%), and accuracy (94.6%/94.7%). In both sets, the panel correctly classified 95% of resistant and 94% of sensitive females. Most compounds identified by the model were lipids and amino acids and revealed pathway alterations related to chemoresistance. Conclusion: We developed a model for predicting patient response to NACT. These metabolite panels allow clinical gain by building precision medicine strategies based on tumor stratification.

## 1. Introduction

Globally, the most common and most lethal type of cancer among women is breast cancer (BC) [1]. This situation, therefore, requires new approaches to improve diagnosis and treatment. This is especially true when we consider the heterogeneous nature of cancer, characterized by complex genetic interactions [2]. In this context, the use of metabolomics as a promising tool for identifying BC biomarkers has achieved great progress and demonstrates future potential for predicting treatment response [3].

Neoadjuvant chemotherapy (NACT) has gained prominence in recent years as an important BC treatment option due to its ability to reduce the cancer burden and promote a pathological complete response (pCR), particularly in BC subtypes that do not express hormone receptors (HRs) [4]. High rates of disease-free survival and overall survival were verified in BC patients with pCR after NACT in comparison to women with residual disease [5]. By classifying BC into subtypes, it is possible to establish the best drugs for treatment. For that, several gene expression markers are used, each identified by immunohistochemistry, including: human epidermal growth factor receptor 2 (HER2), estrogen receptor (ER), progesterone receptor (PR), and nuclear protein Ki67 [6,7,8].

To assess the efficacy of NACT, clinical parameters are mostly used, such as disease response, more specifically described as the residual cancer burden (RCB), a continuous index defined by the size and cellularity of the tumor, in addition to lymph node involvement, evaluating cancer biomarkers in surgical resection specimens by histopathological techniques and applying Cox regression analyses [9]. The RCB index classifies the response to therapy as pathological complete response (pCR) or one of three types of RCB: minimal residual disease (RCBI), moderate residual disease (RCBII), and extensive residual disease (RCBIII) [9]. Various experimental methods aim to enhance the prediction to NACT response and cancer prognosis. These include immunofluorescence, tissue microarrays, DNA/RNA analysis, sequencing, protein quantification, and epigenetic assessments like methylation and cell cycle analysis [2].

Sub-optimal chemotherapy not personalized for patients can lead to various negative outcomes, including resistance to NACT and long-term toxicity (e.g., anthracyclines), which can affect treatment success and increase mortality [10]. HR-positive tumors have lower rates of NACT response and pCR, in contrast to patients with HER2-positive and triple-negative (TN) tumors [11]. Yet, how NACT triggers acquired or innate metabolism resistance still requires extensive investigation [12]. Thus, it is vital to identify new methods for NACT resistance detection so that fewer adverse effects and better responses to therapy can benefit patients [10,13].

In order to add to the well-established tools in the field of omics and advance our understanding of the response of breast tumors to NACT, we developed a method based on the metabolomics of BC. Studies have shown that serum and plasma are suitable samples to the detection of BC metabolites and have provided excellent results in predicting the response to NACT, in addition to being less invasive and more accessible samples than biopsy tissues [14,15,16]. Consequently, using these types of samples, our previous study using nuclear magnetic resonance (NMR) spectroscopy indicated that the amino acids leucine, formate, valine, and proline, as metabolites, were discriminants of NACT response [17]. Also, Ingram et al. found that certain lipids, such as phosphatidylcholine (PC), oxidized lipid species, phosphatidylethanolamine (PE), and sphingomyelin (SM), were present at higher levels in the lipidomic profile of docetaxel-resistant cells (PC3-Rx and DU145-DR) as compared to parent control cells [18].

Therefore, using metabolomic analysis, we aimed to identify plasma metabolite profiles in females with BC before NACT. Then, we compared these profiles to the clinical data obtained after NACT and evidenced alterations in metabolites that could possibly influence tumor response to therapy. As a novelty, the overall aim was to establish a classification model to predict NACT response using pretreatment plasma samples. This would allow easy and relatively rapid assessment of resistance or sensitivity biomarkers to NACT in BC patients, impacting clinical decision making.

## 2. Materials and Methods

### 2.1. Experimental Design

Plasma samples were collected from participating females with invasive ductal carcinoma before any treatment intervention. All participants underwent NACT followed by surgery. Molecular subtyping and outcome classification of participants as sensitive or resistant to NACT was recorded after the surgical removal of the breast tumor following standard protocols. Metabolomic analysis was performed on extracted pre-NACT plasma samples analyzed by liquid chromatography–mass spectrometry (LC-MS). Participants’ clinical and demographic details were collected to be analyzed together with the untargeted metabolomic data. Statistical analysis for this dataset aimed to classify participants as sensitive or resistant to chemotherapy based on their metabolome profile.

### 2.2. Participants, Samples, and Ethical Concerns

Participant female patients were diagnosed and treated at the CAISM Women’s Hospital (Hospital da Mulher Professor José Aristodemo Pinotti, Centro de Atenção Integral à Saúde da Mulher—CAISM), University of Campinas (UNICAMP), Brazil. Approval was obtained from the Institutional Review Board (protocol number 69699717.0.0000.5404). All study participants signed a consent form and were fully informed of their rights to privacy before their biological samples were collected and stored in the CAISM Biobank (CONEP #56, Brazil). This study abides by the Declaration of Helsinki principles.

Plasma and tissue (biopsy) samples were collected between January 2017 and January 2019 from participants with invasive ductal carcinoma (IDC) who underwent NACT followed by surgery (*n* = 75). Peripheral blood and biopsy samples were obtained from participants before they initiated NACT. All participants underwent surgical treatment after NACT, with total mastectomy or quadrantectomy and sentinel lymph node biopsy or axillary lymph node dissection.

### 2.3. Histopathology and Immunohistochemical Evaluation of Tissues

Tissue samples collected from biopsy (pre-treatment specimens) were formalin-fixed and paraffin-embedded. Sections were then hematoxylin-eosin (H&E) stained and reviewed for histologic diagnosis, according to the criteria of the World Health Organization (WHO) [19]. To address tumor heterogeneity, in HR-negative and/or HER2-negative cases, as determined in the pre-treatment tissue samples by immunohistochemistry, the respective surgical specimens with residual disease were assessed again for subtype confirmation [20].

For the classification of BC subtype, the standard immunohistochemical technique was used. The following primary antibodies were applied: anti-estrogen receptor (ER, clone 1D5, 1:1000 *v*/*v*), anti-progesterone receptor (PR, clone PR636, 1:800 *v*/*v*), anti-HER2 (clone PN2A, 1:1100 *v*/*v*), and proliferation marker Ki67 (clone MIB1, 1:500 *v*/*v*). The Envision Flex system and the used antibodies were provided by Dako (Agilent, Santa Clara, CA, USA). The protocol for ER and PR evaluation on the pre-treatment specimens was performed according to Allison et al. (2020) [21]. Cases were considered positive for these receptors if 1% or more of the tumor cells were stained. The Ki67 percentage was obtained by averaging the number of stained tumor cells out of the minimum of 500 total cells observed in different microscopic fields [22]. For HER2 scoring, the recommendations from the American Society of Clinical Oncology/College of American Pathologists (ASCO/CAP) were followed: 0 /1+ cases were considered HER2-negative; 2+ was categorized as equivocal and fluorescence in situ hybridization (FISH) was performed in these cases to confirm; and 3+ cases were considered positive for HER2 amplification [23]. Surgical specimens presenting residual disease were reassessed by immunohistochemistry for subtype confirmation of HR-negative and/or HER2-negative cases, due to tumor heterogeneity [24].

### 2.4. Response to Neoadjuvant Chemotherapy and Outcome Evaluation

The neoadjuvant treatment scheme was clinically indicated according to BC subtype based on the biopsy examination. RCB was used to evaluate NACT response, assessing detailed quantification of residual disease, in a reproducible and fully validated way with long-term follow-up information [9]. RCB provides the final tumor residual dimensions (in mm), quantity of cancer cells contained in the tumor residual area (in percentage), in situ component proportion, if any, quantity of positive lymph nodes, and the nodal metastasis largest diameter (in mm). Cases were grouped into two main sets for statistical analyses, according to NACT response: (a) pCR and RCB-I cases and (b) RCB-II and RCB-III cases [17,25,26,27].

### 2.5. Clinical and Pathological Data

Clinical and pathological data were retrieved from participants’ records, as follows: age at diagnosis, self-declared ethnicity, age of menarche, menopausal status (premenopausal and postmenopausal), use of hormone therapy, previous pregnancies, births, miscarriages, lactation regardless of the number of pregnancies or duration, smoking, chronic alcoholism, body mass index before NACT, hypertension, diabetes mellitus, hypothyroidism, and breast and/or ovarian cancer family history.

Features describing tissue samples were evaluated according to their grade (Nottingham classification), histological type, biomarker rating (ER, PR, Ki67, and HER2), clinical stage according to tumor size, axillary involvement, and presence of distant metastasis. Additionally, treatment was described as breast surgery (mastectomy or quadrantectomy) or armpit surgery (axillary dissection or sentinel lymph node biopsy), and NACT response was evaluated based on surgical tissue specimens according to RCB guidelines [9,28].

### 2.6. Plasma Samples for Metabolomic Analysis

Blood samples from participants were collected in EDTA tubes before NACT and were centrifuged within 2 h of collection. Plasma samples were then transferred to microtubes and frozen at −80 °C until extraction. After thawing, 150 μL aliquots of plasma were extracted by adding 300 μL of cold isopropanol solution. Afterward, the tubes were vortexed for 60 s, centrifuged (12,879× *g*, 10 min, 4 °C), and the supernatant organic layer (300 μL) was collected. Samples were resuspended in a solution of 150 μL acetonitrile (ACN):H_2_O (1:1, % *v*/*v*).

To obtain a quality control (QC) sample, 25 μL from each resuspended specimen was combined to create a unique QC sample. Deviations in extraction and system stability were controlled by inserting one QC sample after every 10 samples. Moreover, a QC sample was used at the beginning of the experiment to perform instrumental stabilization of the LC-MS system. The order of sample extraction and subsequent analysis was randomized to minimize any potential bias related to instrument or biological variables.

### 2.7. Metabolomic Analysis Using LC-MS

The analyses were previously described by our group [29,30]. An ACQUITY UPLC coupled to an XEVO-G2XS quadruple time-of-flight mass spectrometer (QToF) (Waters, Manchester, United Kingdom) was used, supplied with an electrospray ionization (ESI) source and operated in negative (ESI) ionization mode. An ACQUITY UPLC^®^ CSH C18 column (C18, 2.1 mm × 100 mm × 1.7 μm, Waters) was employed using mobile phase A, with ACN/H_2_O solution (60:40, *v*/*v*) and 10 mM ammonium formate + 0.1% formic acid, and mobile phase B, comprising isopropanol/ACN (90:10, *v*/*v*) with 10 mM ammonium formate + 0.1% formic acid.

The flow rate was 0.4 mL min^−1^. The column was eluted with 40% Solution B, which was increased to 43% over 2 min and then reached 50% within 0.1 min. The gradient was ramped to 54% over the next 9.9 min and to 70% in 0.1 min. The amount was finally increased to 99% over 5.9 min. The concentration of Solution B finally returned to 40% in 0.1 min, and the column was balanced for 1.9 min before the next injection. The total run time was 20 min and the injection volume was 1 μL.

Data were obtained in MS^E^ mode employing 6 V for low collision energy and a ramp of 20–50 V for high collision energy, with the *m/z* range set from 50 to 1200 Da and 0.5 s for scan duration. Leucine enkephalin (molecular weight = 555.62; 200 pg L^−1^ in 1:1 ACN:H_2_O, *v*/*v*) was used as the lock mass, and calibration was performed using 0.5 mM sodium formate solution. Additional settings included: source temperature of 140 °C, desolvation temperature of 550 °C, desolvation gas flow rate of 900 L h^−1^, capillary voltage of 2.5 kV, and cone voltage of 40 V.

### 2.8. Data Pre-Processing

Raw LC-MS data were analyzed using Progenesis™ QI software version 2.4 (Nonlinear Dynamics in Newcastle, United Kingdom). This software facilitated the identification of potential adducts, as well as the alignment of peaks, deconvolution process, and annotation of compounds, all based on MS^E^ experiments. The adducts [M-H]^−^, [M + Cl]^−^, [M-H_2_O-H]^−^, and [M + FA-H]^−^ were taken into account. An intensity table of ions was generated by Progenesis QI for each sample, labeled according to their nominal masses and retention time, in agreement with their intensity (areas of the extracted ion chromatogram), which are called ‘features’. Ion abundance data were corrected by the QC pool using the QC-based random forest signal correction (QC-RFSC) method implemented in Systematic Error Removal using the Random Forest (SERRF) package [31]. Missing values were replaced by the minimum value ± random error of the dataset. Afterward, relative standard deviation (%RSD) was determined for the QC samples.

### 2.9. Statistical Analysis of the LC-MS Data

The MetaboAnalyst™ 6.0 modules Statistical Analysis and Biomarker Analysis were also used for data analysis [32]. In summary, when the dataset was uploaded, only features with a %RSD < 10% in the QC samples were kept, median-normalized, log-transformed, and scaled by Pareto. The volcano plot served as an instrument for selecting features, employing criteria such as a *p*-value of the *t*-test < 0.05 and a fold change of ±1.5. Furthermore, significant features were used to build a partial least square–discriminant analysis (PLS-DA), and those features with a score of variables in projection (VIP) > 1 were kept to be used in the classification model. The score plots for principal component analysis (PCA) were used to visualize data clustering before and after feature selection. The classification model was created using a support vector machine (SVM) and was evaluated based on sensitivity, specificity, accuracy, area under the receiver operator characteristics curve (AUC), negative predictive value (NPV), and positive predictive value (PPV).

### 2.10. Putative Identification of Metabolites and Pathway Enrichment Analysis

Metabolite identification was based on MS^E^ analysis. Because of the acquisition of low and high energy in the same spectrum, information was collected for precursor ions (lower energies; mass error ≤ 5) and fragments (higher energies; tolerance ≤ 10 ppm). We also evaluated the fragmentation score, mass error, mass accuracy, and isotope similarity against the annotated molecules [33,34]. The compatibility of Progenesis QI data and external SDF-based spectra libraries was enabled using in-house software, SDF2PQI, to increase fragment matches [35]. SDF2PQI has been detailed in a freely available and open-source publication (https://github.com/pedrohgodoys/sdf_to_pqi, accessed on 15 April 2024). LipidMaps [36], Human Metabolome Database [37], and MassBank of North America (MoNA) were used as external SDF-based spectral libraries [38]. The identified metabolites were used for pathway enrichment analysis in the Reactome platform [39]. Pathways were selected based on a false discovery rate (FDR) < 0.05.

### 2.11. Evaluation of Classification Bias via Cross-Validation with Permuted Data

To address potential overfitting of the classification model, we evaluated the robustness of the SVM-based approach using 10-fold cross-validation with multiple replications. The evaluation was performed on the significant features selected by PLS-DA with VIP > 1, which were detected in the identification step. Specifically, we divided the complete metabolite dataset (samples × metabolites) into 10 equal folds. Moreover, to further investigate potential biases in the data, we created 100 datasets by permuting the sample class labels and applied the same cross-validation method described earlier, performing 10 replications for each dataset. Cross-validation and sampling of replications were performed using wrapper functions available in the R package MetabolomicsBasics using the parameters *n* = 1000, k = 10, and n_rand = 10. For the observed data model and the permuted model, the metrics accuracy, specificity, and sensitivity were analyzed [40].

## 3. Results

### 3.1. Clinical and Pathological Data

Table 1 shows the demographic, clinical, and pathological tumor features, distributed according to sensitivity or resistance to NACT, based on pCR or RCB classification. All participants had invasive ductal carcinoma, among which 51% presented tumors of histological grade 3, and 59% were HR-positive. Participants with histological grade 3 tumors had a lower probability of being sensitive, i.e., pCR/RCB-I (OR = 0.16 (0.04–0.62); *p* = 0.0046) than the participants whose tumors were grade 1 or 2. Also, participants with HR-positive tumors were less prone to being sensitive (OR = 6.3 (1.9–20.9); *p* = 0.0033) than participants with non-luminal tumors.

The distribution of NACT response regimens was also analyzed (Table S1), highlighting that sensitivity to NACT was demonstrated by 62.5% of HER2+ subtype and 36.4% of TN subtype tumors. Luminal tumors did not show high response rates, with resistance rates varying from 66.6% to 100% depending on the regimen. These results are in agreement with the majority of clinical studies performed in recent years [13].

### 3.2. Detection of Metabolites Related to NACT Resistance

After LC-MS detection of 7066 features, a volcano plot followed by analysis of VIP scores from PLS-DA pointed to 60 statistically significant features. From these, 19 features were identified, as shown in Table 2. PLS-DA score plots were obtained for the identified features, which are displayed in Figure S1. The percentage of features identified was 20%, which is in agreement with the expected rate of identification [41].

**Figure 1:**
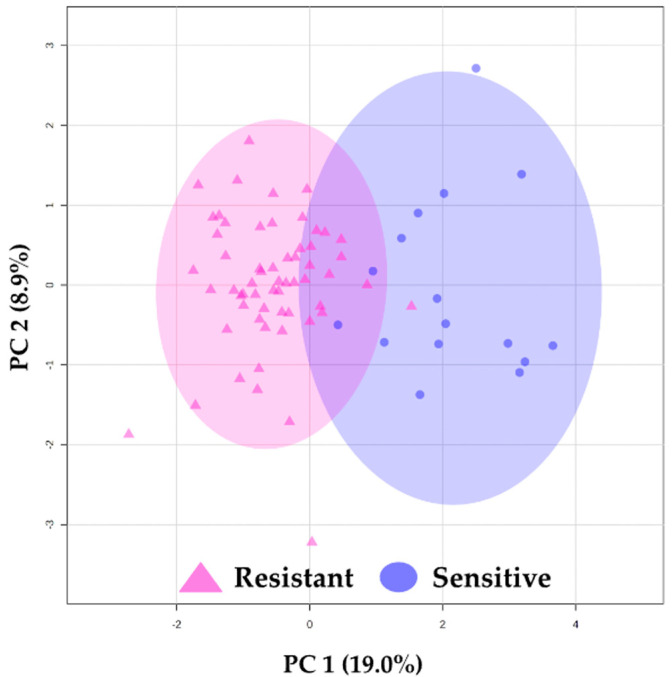
Principal component analysis (PCA) results. PCA using 19 identified features. △ (pink) represents resistant plasma samples, and ◯ (blue) represents sensitive plasma samples. The ellipses indicate the confidence interval (CI = 95%).

To demonstrate the potential of these metabolites to differentiate between sensitive and resistant sample groups, we used a PCA score plot after feature selection (Figure 1), which showcased a trend toward the separation between sensitive and resistant samples.

Among the 19 identified compounds presented in the model (Table 2), 9 were lipids, classified into glycerophospholipids (*n* = 7) and fatty acyls (*n* = 2), while other important metabolites involved in NACT response discrimination were amino acids (*n* = 9) and bile acids and derivatives (*n* = 1). All identified compounds and their statistical parameters are listed in Table S2.

### 3.3. Prediction of Response to NACT

For creating a classification model, we used SVM to discriminate NACT-resistant samples from NACT-sensitive ones. Only the 19 identified features were used to compose the model, which was built based on a training set of 75% (resistant *n* = 44, sensitive *n* = 12) and tested with a validation set of 25% (resistant *n* = 15, sensitive *n* = 4).

**Figure 2:**
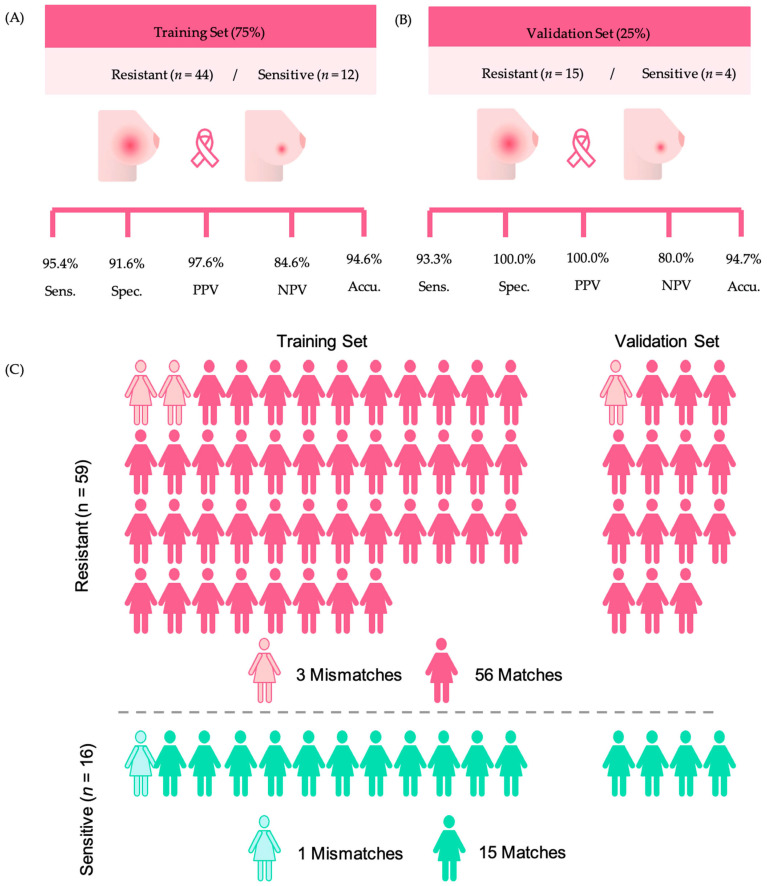
Results obtained with the SVM model, considering resistant and sensitive samples. (**A**) Training set sensitivity, specificity, accuracy, negative predictive value (NPV), and positive predictive value (PPV). (**B**) Validation set sensitivity, specificity, accuracy, NPV, and PPV. (**C**) Representation of the model’s ability to make predictions of NACT response.

The training set of the SVM model presented 95.4% sensitivity, 91.6% specificity, 94.6% accuracy, 97.6% PPV, 84.6% NPV (Figure 2A), and average AUC of 0.969, showing great potential for clinical use. To further investigate the predictive power of the model in differentiating NACT response, the SVM algorithm was used to classify the validation sample set composed of resistant (*n* = 15) and sensitive (*n* = 4) samples. The SVM performance of this model is displayed in Figure 2B. Remarkably, among the 19 samples that comprised the validation set, only one HER2+ sample was misclassified by the model, resulting in 93.3% sensitivity, 100.0% specificity, 80.0% NPV, 100.0% PPV, and an accuracy of 94.7% (Figure 2B).

Overall, when considering the complete group of NACT-resistant samples, regardless of their use in the training or validation sets, the metabolite panel correctly classified 56/59 (94.9%) of the tumors as resistant to chemotherapy (Figure 2C). Similarly, the model correctly classified 15/16 (93.7%) of the tumors as sensitive to NACT. The detailed information on the matches of the model across sensitivity or resistance and their use as training or validation sets are displayed in the supporting Table S3.

To evaluate the reliability of the SVM model, which is susceptible to overfitting, we conducted a 10-fold cross-validation across numerous iterations. Specifically, we divided a comprehensive metabolite dataset (75 samples, 19 metabolites) into ten equal parts. We then used nine parts as training data to predict the class labels of the remaining part, repeating this process to assess overall prediction accuracy. This method was replicated 1000 times, each with a unique division of the dataset into ten parts. In this way, we obtained a robust average prediction accuracy of 94.2%, sensitivity of 95.4%, and specificity of 87.0%, while for permuted data, the resulting average prediction accuracy was 70.2%, sensitivity was 82.3%, and specificity was 11.1% for the SVM model, as seen in Figure S2.

### 3.4. Pathway Analysis

The identified metabolites from resistant versus sensitive sets were used to perform pathway enrichment analysis in the Reactome platform, which resulted in 45 pathways found to be probably related to NACT resistance with an FDR < 0.05. Thus, we selected the pathways that were most related to carcinogenicity and chemoresistance process for discussion, which were: amino acid transport across the plasma membrane (FDR: 3.53 × 10^−6^); disorders of transmembrane transporters (FDR: 2.06 × 10^−4^); MAPK/MAPK3 signaling (FDR: 4.75 × 10^−3^); SLC transporter disorders (FDR: 1.10 × 10^−3^); SLC-mediated transmembrane transport (FDR: 2.4 × 10^−3^); ERBB2 signaling pathway (FDR: 2.09 × 10^−2^); and plasma lipoprotein assembly, remodeling, and clearance (4.13 × 10^−2^). The complete list of pathways containing the number of identified metabolites for each pathway can be seen in Table S4.

## 4. Discussion

Regarding NACT outcome prediction, our aim was to develop a useful panel to identify BC patients who would not benefit from NACT, potentially avoiding their unnecessary exposure to toxic chemotherapy drugs. We modeled an untargeted panel of metabolites retrieved from plasma samples of 75 female participants with BC to unveil the ability to predict NACT resistance. The panel showed outstanding clinical application potential to identify metabolites. Using a panel of 19 identified compounds, we achieved effective prediction of NACT response, demonstrating high sensitivity (93.3%), specificity (100%), and accuracy (94.7%).

Multigene panels now serve as an effective tool for distinguishing between tumor subgroups, identifying those that are most responsive or inherently resistant to existing pharmacological treatments, although there is a lack of tests that can accurately predict the response to neoadjuvant therapy [42]. It is known that NACT response, when not targeted to specific tumor subgroups, especially for luminal-type breast tumors, is rather low [10,13,43]. Therefore, finding ways to improve response prediction is essential. The simple and relatively fast method used in the present study to extract plasma was based on protein precipitation, and it presented good quality metrics. The subsequent analysis using high-resolution mass spectrometry allowed the identification of compounds in the plasma of females with BC that have the potential to be blood-based biomarkers of NACT response, consequently enabling more successful treatment.

The developed model misclassified three samples in the training group (two resistant and one sensitive) and one sample (resistant) in the validation group. We infer that factors such as sample classification into subtypes or assessment of RCB influenced the misclassification. There might be genetic particularities in these plasma samples that were not detected by the biomarkers assessed, or there could be residual tumor cells not identified by our immunohistochemistry methods, a known restraint of this technique [44]. Furthermore, several other studies have shown that, despite limitations, metabolite panels are a viable and promising option for predicting the response to therapies in BC [15,17,45,46,47,48,49,50,51], colorectal cancer [52,53,54], cervical cancer [55], esophageal tumors [56], and pancreatic cancer [57].

Our study highlighted key lipid metabolites related to chemoresistance, which is crucial to better understand the role of lipids in drug resistance. Lipid metabolism is known for its implications in BC development and progression, influencing tumor behavior and therapy response [58,59,60]. Drug resistance has been linked to augmented fatty acid synthesis and changes in cell composition, for example, in the spatial distribution and fluidity of sphingolipids and cholesterol found in lipid rafts [61]. Also, some lipid pathways that affect fatty acid metabolism, such as lipid biosynthesis, desaturation, droplet formation, and catabolism, contribute to malignant tumor growth, metastasis, and chemoresistance [62,63,64,65]. The lipid pathway shown in our study was related to “plasma lipoprotein assembly, remodeling and clearance”. For instance, high levels of certain plasma lipoproteins have been linked to an increased risk of BC, suggesting that lipid peroxidation and oxidative stress may be involved in disease pathology [66]. Also, nine lipids were found to have the potential to differentiate sensitive and resistant patients (PC 20:3/18:1, PC 20:3/16:0, PC 18:0/22:4, FA 18:1;O2, DG 20:1/0:0/20:4, PE 20:4/22:0, PE 18:0/18:3, PE PGD1/22:5, and PE-NMe2 20:0/14:0). In particular, we highlight phosphatidylethanolamines (PEs), which were previously related to BC aggressiveness [67] and are known for being an important component of biological membranes [68].

In addition to the role of lipids in differentiating chemoresistant patients, amino acids, as the second most abundant class of compounds found in our analysis, were also shown to play an essential role in some pathways, such as “amino acid transport across the plasma membrane” and “disorders of transmembrane transporters”. Recently, amino acids and altered amino acid metabolism were associated with BC chemoresistance [69]. The L-type amino acid transporter 1 (*LAT1*) was found to promote chemoresistance in ER-positive/HER2-negative breast cancer [69] by facilitating the uptake of other amino acids, which can be used by cells for energy production and biomass synthesis, supporting the growth of cancer cells. This happens especially for branched-chain or aromatic amino acids, such as leucine, isoleucine, valine, phenylalanine, tyrosine, tryptophan, methionine, and histidine [70]. *LAT1* expression levels correlated with cell proliferation after chemotherapy, indicating its role in treatment resistance, particularly in the luminal BC subtype [69]. Interestingly, leucine, tryptophan, and histidine, related to *LAT1* uptake, were also indicated in our previous study as being discriminant between sensitive and chemoresistant patients when using NMR-based metabolomics to investigate a similar set of samples [17]. Consequently, variations in amino acid distribution may enhance treatment selection when deciding whether or not to use NACT for BC.

Several molecular mechanisms are associated with multidrug resistance in BC. One of the most significant mechanisms involves efflux transporters, specifically ATP-binding cassette (ABC) transporters. These transporters use ATP to actively pump chemotherapeutic drugs out of cancer cells, thereby reducing intracellular drug accumulation and contributing to chemoresistance [71,72]. In fact, our findings identified the “disorders of transmembrane transporters” pathway, which is likely attributed to the involvement of efflux transporters and their association with chemoresistance. Also among our findings, we identified the pathway “MAPK/MAPK3 signaling”, which is involved in promoting cell survival and chemotherapy response by the tumor [73].

Our findings also encompassed the “ERBB2 signaling pathway”, with this BC driver gene being responsible for coding HER2, a transmembrane glycoprotein that belongs to the epidermal growth factor receptor (EGFR) family [74]. HER2 overexpression is associated with aggressive BC phenotypes and increased chemoresistance to certain chemotherapeutic agents [75] due to downstream signaling pathways that promote cell survival and proliferation [74,76].

“G-protein mediated events” was also listed among the impacted pathways. G protein-coupled receptors (GPCRs) constitute the largest family of cell surface receptors, playing a diverse array of signal transduction pathways and cellular functions, including cell proliferation, survival, and motility as key regulators in tumor growth, angiogenesis, and metastasis [77]. Therefore, emphasizing GPCR as a therapeutic target is crucial for new treatment approaches, underscored by the large range of medications available that focus on these receptors [78].

Our study revealed two pathways related to solute carriers (SLCs): “SLC transporter disorders” and “SLC-mediated transmembrane transport”. The SLC superfamily is crucial in both the development and treatment of BC [79]. These transporters, embedded within cellular membranes, are responsible for the movement of various substances, such as nutrients, ions, and drugs. When SLC transporter disorders occur, it can disrupt the processing of natural compounds like estrogen, essential in certain types of BC [79,80]. In relation to chemoresistance, SLC expression levels can affect how cancer cells respond to drugs, contributing to either sensitivity or resistance.

While our study demonstrates the promising translational potential of MS-based metabolomic panels, it is essential to validate these results in an independent cohort to confirm their reproducibility [81]. Additionally, our study is constrained by a few limitations, including the relatively small number of participants, the low incidence of positive treatment response, and the heterogeneous nature of tumor types under investigation. Nonetheless, the approach we used to filter molecular signatures from irrelevant chemical noise could help in establishing more robust panels, and this should promote a real clinical gain based on MS-based metabolomics.

## 5. Conclusions

In conclusion, we developed a fast and effective analytical model for predicting BC patient response to NACT using blood-based biomarkers. This model contributes to providing a reliable prediction of BC response to NACT before treatment commences and highlights crucial pathways related to chemoresistance. The advantage of using this panel is that non-responsive patients would not be exposed to toxic chemotherapy drugs, leading to better outcomes for them.

## Figures and Tables

**Table 1:** Demographic, clinical, and pathological features of the tumors, distributed according to the pCR/RCB classification.

Features	Classification	n (%)	pCR/RCB I (Sensitive) *n* = 16 (%)	RCB II/III (Resistant) *n* = 59 (%)	OR (95% CI)	*p*-Value
Age at diagnosis	<45	22 (29.3)	6 (37.5)	16 (27.1)	ref	
	≥45	53 (70.6)	10 (62.5)	43 (72.9)	1.61 (0.5–5.16)	0.44
Ethnicity	Caucasian	64 (85.3)	15 (93.7)	49 (83)	ref	
	Non-Caucasian	11 (14.6)	1 (6.3)	10 (17)	3.06 (0.36–25.9)	0.32
Age at menarche	<12	17 (22.6)	3 (18.7)	14 (23.8)	ref	
	≥12	58 (77.3)	13 (81.3)	45 (76.2)	0.74 (0.18–2.98)	0.71
Pregnancy *	Yes	69 (92.0)	15 (93.7)	54 (91.5)	ref	
	No	6 (8.0)	1 (6.3)	5 (8.5)	1.39 (0.15–12.81)	0.85
Lactation **	Yes	60 (87.0)	14 (87.5)	46 (77.9)	ref	
	No	9 (13.0)	2 (12.5)	7 (22.1)	1.07 (0.2–5.72)	0.98
Menopause	Yes	42 (56.0%)	7 (43.7)	35 (59.3)	ref	
	No	33 (44.0%)	9 (56.2)	24 (40.7)	0.53 (0.17–1.63)	0.28
Hormone therapy	Yes	12 (16.0)	1 (6.3)	11 (18.6)	ref	
	No	63 (84.0)	15 (93.7)	48 (81.4)	0.29 (0.03–2.44)	0.26
Family history (breast and ovarian cancer)	Yes	19 (25.3)	7 (43.7)	12 (20.3)	ref	
	No	56 (74.6)	9 (56.2)	47 (79.7)	3.05 (0.94–9.85)	0.076
Comorbidities	Obesity (BMI ≥ 30)	30 (40)	7 (43.7)	23 (39.0)	ref	
	Diabetes	9 (12)	1 (6.3)	8 (13.5)	2.43 (0.26–22.97)	0.49
	Hypertension	29 (38.6)	4 (25.0)	25 (42.4)	1.9 (0.49–7.36)	0.37
	Hypothyroidism	8 (10.6)	1 (6.3)	7 (11.9)	2.13 (0.22–20.41)	0.57
Other conditions	Smoking	15 (20)	4 925.0)	11 (18.7)	ref	
	Chronic alcoholism	1 (1.3)	0 (0.0)	1 (1.7)	Inf (NaN-Inf)	0.75
Clinical stage	I/II	47 (62.7)	12 (75.0)	35 (59.3)	ref	
	III/IV	28 (37.3)	4 (25.0)	24 (40.7)	2.06 (0.59–7.15)	0.27
Histological grade	1/2	38 (50.6)	3 (18.7)	35 (59.3)	ref	
	3	37 (49.4)	13 (81.3)	24 (40.7)	0.16 (0.04–0.62)	0.0046
Hormonal receptor	Negative	19 (25.3)	9 (56.2)	10 (16.9)	ref	
	Positive	56 (74.7)	7 (43.7)	49 (83.1)	6.3 (1.9–20.9)	0.0033
Ki67	Low	32 (42.7)	6 (37.5)	26 (44.1)	ref	
	High	43 (57.3)	10 (62.5)	33 (55.9)	0.76 (0.24–2.37)	0.66
Molecular subtype	Luminal HER2−	35 (46.7)	2 (12.5)	33 (56.0)	ref	
	Luminal HER2+	21 (28)	5 (31.2)	16 (27.1)	0.19 (0.03–1.11)	0.07
	Non-Luminal HER2+	8 (10.6)	5 (31.2)	3 (5.1)	0.04 (0–0.27)	0.0011
	Triple-Negative	11 (14.7)	4 (25.0)	7 (11.8)	0.11 (0.02–0.7)	0.025

Hormonal receptor: positive if ER- and/or PR-positive; negative if both ER- and PR-negative. BC patients without comorbidities or other conditions were not considered. * Two participants had abortions in all pregnancies. ** Six participants had no pregnancies, so they did not breastfeed. pCR, pathological complete response; RCB, residual cancer burden; OR, odds ratio; CI, confidence interval; BMI, body mass index; Ki67, Ki67 protein; HER2, human epidermal growth factor receptor.

**Table 2:** Significant metabolites identified and used for building the predictive model.

Feature	Adducts	Formula	Description	Mass Error (ppm)	Trend in Resistant Samples
1.45_481.3518 *m*/*z*	M + FA-H	C_27_H_48_O_4_	ST 27:0;O4 ^a^	−3.74	↓
11.15_854.5911 *m*/*z*	M + FA-H	C_46_H_84_NO_8_P	PC 20:3/18:1 ^b^	−3.32	↑
14.61_764.5587 *m*/*z*	M-H_2_O-H	C_44_H_82_NO_8_P	PC 20:3/16:0 ^b^	−1.61	↑
11.24_898.5038 *m*/*z*	M + Cl	C_47_H_78_NO_11_P	PE PGD1/22:5 ^b^	3.62	↓
11.57_792.5728 *m*/*z*	M + FA-H	C_41_H_82_NO_8_P	PE-NMe2 20:0/14:0 ^b^	−4.33	↑
8.87_715.5531 *m*/*z*	M + FA-H	C_43_H_74_O_5_	DG 20:1/0:0/20:4 ^c^	1.95	↓
14.20_804.5913 *m*/*z*	M-H2O-H	C_47_H_86_NO_8_P	PE 20:4/22:0 ^b^	0.03	↓
10.12_740.5212 *m*/*z*	M-H	C_41_H_76_NO_8_P	PE 18:0/18:3 ^b^	−3.21	↓
13.43_882.6218 *m*/*z*	M + FA-H	C_48_H_88_NO_8_P	PC 18:0/22:4 ^b^	−1.36	↑
0.53_187.0721 *m*/*z*	M-H	C_7_H_12_N_2_O_4_	N-Acetylglutamine ^d^	−1.89	↑
0.56_203.0818 *m*/*z*	M-H	C_11_H_12_N_2_O_2_	L-Tryptophan ^d^	−3.94	↓
0.82_313.2372 *m*/*z*	M-H	C_18_H_34_O_4_	FA 18:1;O2 ^c^	−3.87	↓
0.56_130.0871 *m*/*z*	M-H	C_6_H_13_NO_2_	L-Leucine ^d^	−1.58	↑
0.53_195.0503 *m*/*z*	M-H	C_6_H_12_O_7_	Gluconic acid ^d^	−3.6	↑
1.96_168.0304 *m*/*z*	M-H	C_7_H_7_NO_4_	2-Furoylglycine ^d^	0.78	↑
0.54_124.0076 *m*/*z*	M-H	C_2_H_7_NO_3_S	Taurine ^d^	1.49	↓
0.63_154.0618 *m*/*z*	M-H	C_6_H_9_N_3_O	L-Histidine ^d^	−2.51	↑
0.56_114.0555 *m*/*z*	M-H	C_5_H_9_NO_2_	L-Proline ^d^	−4.97	↑
8.68_128.0352 *m*/*z*	M-H_2_O-H	C_5_H_9_NO_4_	L-Glutamic acid ^d^	−0.88	↓

Legend: ^a^ Bile acids and derivatives; ^b^ Glycerophospholipids; ^c^ Fatty Acyls; ^d^ Amino acids and derivatives; ↓ downregulated in resistant samples; ↑ upregulated in resistant samples.

## Data Availability

All metabolomic data are deposited in Metabolights under identifier MTBLS6797.

## Supplementary Materials

The following supporting information can be downloaded at: https://www.mdpi.com/article/10.3390/cancers16132473/s1, Figure S1: Partial Least-Squares Discriminant Analysis (PLS-DA) results with cross-validation metrics; Figure S2: Histograms of accuracy, sensitivity, and specificity from 1000 replications of observed and permuted data in 10-fold cross-validation to evaluate the robustness of the SVM classification model. To permute the data, class labels were randomly assigned, and replications indicated various potential splits in cross-validation. Table S1: Distribution of response according to the NACT therapeutic regimen; Table S2: Attributed compounds to the 19 features used for model establishment; Table S3: Detailed information of the samples used to predict NACT sensitivity or resistance, Table S4: Detailed pathways impacted.
